# The Utility of Neuromuscular Assessment to Identify ADHD Among Patients with a Complex Symptom Picture

**DOI:** 10.1177/10870547241273102

**Published:** 2024-09-02

**Authors:** Anne Beate Helseth Udal, Liv Larsen Stray, Are Hugo Pripp, Torstein Stray, Jens Egeland

**Affiliations:** 1 Department of Psychiatry, Sørlandet Hospital HF, Kristiansand, Norway; 2 Department of Biostatistics, Oslo University Hospital, Norway; 3 Department of psychology, University of Oslo, Norway; 4 Vestfold Hospital Trust, Tønsberg, Norway

**Keywords:** adult ADHD, motor control, diagnosis, comorbidity

## Abstract

**Objective:**

Diagnostic assessment of ADHD is challenging due to comorbid psychopathologies and symptoms overlapping with other psychiatric disorders. In this study, we investigate if a distinct pattern of neuromuscular dysregulation previously reported in ADHD, can help identifying ADHD in psychiatric patients with diverse and complex symptoms.

**Method:**

We explored the impact of neuromuscular dysregulation, as measured by The Motor Function Neurologic Assessment (MFNU), on the likelihood of being diagnosed with ADHD, affective disorder, anxiety disorder, or personality disorder among adults (*n* = 115) referred to a psychiatric outpatient clinic.

**Results:**

Logistic regression revealed that neuromuscular dysregulation was significantly associated with ADHD diagnosis only (*OR* 1.15, *p* < .01), and not with affective-, anxiety-, or personality disorders. Sensitivity and specificity for ADHD at different MFNU scores is provided.

**Conclusions:**

A test of neuromuscular dysregulation may promote diagnostic accuracy in differentiating ADHD from other psychiatric disorders in patients with an overlapping symptom picture. This may have important implications for clinical practice. More studies are needed.

## Background

Attention deficit hyperactivity disorder (ADHD) is a childhood onset neurodevelopmental disorder characterized by a persistent and pervasive pattern of cognitive and behavioral dysregulation, negatively affecting personal, academic, and social functioning (American Psychiatric Association [APA], 2013). Persistent adult ADHD (diagnosed in childhood) affects roughly 2.5% of the adult population globally (Faraone et al., 2021), whereas the prevalence of adult ADHD regardless of childhood diagnosis is about 6.8%. (Song et al., 2021). There are significant discrepancies in reported prevalence due to differing classification systems and diagnostic practice, and dimensionally distributed symptoms overlapping with symptoms of other psychiatric disorders (Katzman et al., 2017; Polanczyk & Jensen, 2008). While debates persist regarding the validity of adult ADHD as a childhood onset disorder (Moffitt et al., 2015), both family studies, neuroimaging studies and similar stimulant effects in children and adults suggest that childhood ADHD and adult ADHD are manifestations of the same disorder, despite fewer brain findings and a wider range of symptoms in adults (Zhang-James et al., 2021). Adult ADHD is often characterized by symptoms related to executive dysfunction, inner restlessness, and emotional lability that may be misinterpreted as affective, anxiety-, or personality disorders (Asherson, 2005; Franke et al., 2018; Katzman et al., 2017; J. J. Kooij et al., 2012). Besides, ADHD is often comorbid with other psychiatric disorders, above all affective-, anxiety-, personality- and substance use disorders (Choi et al., 2022), which may be the presenting problems by referral to specialist health services (Pehlivanidis et al., 2014). This complicates the clinical picture and may result in misdiagnosis, ineffective treatment, impaired function, and poor long-term outcome (Asherson et al., 2012; Halmoy et al., 2009; Shaw et al., 2012; Sobanski, 2006).

The bulk of evidence suggests that ADHD is underdiagnosed in adults (Ginsberg et al., 2014). A European, multinational study of 5,662 adult psychiatric outpatients, revealed a mean ADHD prevalence of 15.8% and even more in Northern Europe (30.7%). The patients with previously unrecognized ADHD (53.9%) had more comorbidities and functional impairments than those with previously diagnosed ADHD (Deberdt et al., 2015). This highlights the necessity of developing assessment tools that can facilitate more dependable ADHD diagnostics.

Considerable research effort has been devoted to identifying potential neurobiological markers of ADHD. One area of research has focused on dopamine dysregulation, which has been shown to be important in the neurobiology of ADHD (Curatolo et al., 2010; Kasparek et al., 2015). Neuroimaging studies of ADHD indicates reduced connectivity in dopaminergic fronto-striato-cerebellar circuits of the brain (Castellanos & Proal, 2012; Curatolo et al., 2010; De La Fuente et al., 2013). These circuits exert strong modulatory effect not only on attention and executive functions, but also on mood- and motor regulation. The midbrain dopaminergic system (a part of the reticular activating system), is also involved in cognitive and motor control, both attention, arousal, ability to focus, and modulation of muscle tone (Arguinchona & Tadi, 2022; Urbano et al., 2017). Sagvolden et al. (2005) argue that difficulties that have previously been allocated to a dysfunction in the prefrontal cortical areas controlling executive functions (Barkley et al., 2001), may be better accounted for as hypofunction in dopaminergic circuits. Dopamine is a key neurotransmitter involved in both higher cognitive functions and motor control symptoms, among others (Speranza et al., 2021). Methylphenidate (MPH), the first-choice pharmacological treatment of ADHD, increases dopamine signaling through multiple actions and may improve ADHD-related brain abnormalities (Kasparek et al., 2015; Schweren et al., 2013). A positive effect of MPH on postural stability, motor timing, and other motor problems in individuals with ADHD has been reported by several researchers (Jacobi-Polishook et al., 2009; Rubia et al., 2009; Shorer et al., 2013; Stray, Stray, Iversen, Ruud, & Ellertsen, 2009). The association of motor problems and ADHD, has been known for decades (Harvey & Reid, 2003). The previous used diagnosis “Minimal Brain Dysfunction (MBD)” included motor deficits as one of the problem components (Gillberg & Kadesjo, 2003). The removal of motor problems from the inclusion criteria of ADHD may have led to a decline in international interest in these issues in ADHD.

Motor problems in the form of DCD co-occur in approximately 50% of ADHD cases (Pranjic et al., 2023). *DCD* is a common neurodevelopmental disorder characterized by serious impairment in the acquisition and execution of motor skills and related coordination that negatively impact on activities of daily living (APA, 2013). The motor problems continues into adulthood in most cases (Purcell et al., 2015). Other motor problems commonly observed in ADHD include impaired motor timing, inhibition problems, balance problems, motor overflow, and increased muscle tone (D’Agati et al., 2010; Fliers et al., 2008; Noreika et al., 2013; Stray et al., 2013; Udal et al., 2009). In the differential diagnoses section for DCD in the DSM-IV manual (APA, 2000) it is stated that “Individuals with Attention-Deﬁcit-Hyperactivity Disorder may fall, bump into things, or knock things over, but this is usually due to distractibility and impulsiveness, rather than to a motor impairment.” Nonetheless, empirical evidence consistently indicates that while inattention and impulsivity may contribute to instances of “ADHD clumsiness” genuine motor deficits frequently underlie these manifestations, particularly evident in tasks requiring fine motor skills (Pitcher et al., 2003). Research has revealed that movements in children with ADHD often exhibit increased jerkiness and require more time to change direction of movement compared to controls (Eliasson et al., 2004; Yan, 2002). Children at risk for ADHD are reported to be generally less accurate and more variable in their movements relative to those with other psychopathologies or controls without ADHD (Kalff, 2003). A recent study found that impaired motor coordination and visual perception was the only test-performance predictors of retained ADHD diagnosis after 25 years (Oie et al., 2023). It has been argued that motor problems in ADHD seem related to neuromuscular dysregulation, rather than to deficits in motor skills (acquired voluntary control over movements) as measured by standard motor tests batteries (Stray et al., 2015). The term “neuromuscular regulation” refers to the complex interplay between the neural system and the musculoskeletal system (Röhrle et al., 2019). Stray and her different research teams from 1998 to 2013 (see Stray et al., 2015) have consistently found that the deficiencies in motor function in persons with ADHD are specifically related to dysfunctions in two specific (but probably interlinked) facets of neuromotor regulation, that is problems with *muscular inhibition*, and a *heightened muscle tone* in the gross movement muscles of the back, shoulders, hips, and legs. These deficits may not consistently manifest when utilizing standardized motor tests such as the Movement Assessment Battery for Children (MABC) (Henderson & Sugden, 1992) or comprehensive neuropsychological test batteries like the Halstead/Reitan battery (Reitan, 1985). The research work resulted in the development of the Motor Function Neurological Assessment battery (MFNU) (Stray et al., 2006), which specifically targets these deficiencies. Using the MFNU, both motor dysregulation and a heightened muscle tone, was discovered in close to 90% of both children and adults with ADHD, whereas normal controls presented very few such problems (Stray et al., 2013; Stray, Stray, Iversen, Ruud, Ellertsen, & Tonnessen, 2009). For adults, a median MFNU score of 25 (of max. 32) for the ADHD group, and 2 for the controls was obtained. In a study of children with ADHD, those who responded well to the MPH treatment on the core symptoms of ADHD, showed significantly more pre-treatment motor dysregulation as assessed by the MFNU, than the non-responder group (Stray et al., 2010). A double-blind study performed by the same research team, showed that motor dysregulation improved significantly with an acute dose of MPH, and returned undiminished when the MPH was metabolized (Stray, Stray, Iversen, Ruud, & Ellertsen, 2009); video examples of MPH response on muscular regulation can be seen in additional files.

The beneficial stimulant effects on core symptoms of ADHD is shown to be concomitantly associated with normalization of the neuromuscular problems in persons with ADHD (Stray, Stray, Iversen, Ruud, & Ellertsen, 2009). This demonstrates a possible functional parallelism between the core symptoms of ADHD and the neuromuscular regulation problems. Thus, an evaluation of neuromuscular regulation problems may prove useful as a clinical sign of ADHD.

However, it is quite possible that neuromuscular regulation problems might be found in other clinical groups as well. It is therefore premature to conclude that the MFNU-results may be a clinical marker of ADHD alone. Notably, the conclusions drawn from the above studies were derived from a contrast of individuals diagnosed with ADHD and normal controls. Thus far, there is a lack of research examining the incidence of neuromuscular dysregulation problems within a wider psychiatric populations. The present study is a first step to clarify the specificity of the association between neuromuscular regulation problems, as defined by the MFNU, and ADHD. We posed the question: Can the presence of neuromuscular dysregulation be a clinical sign of ADHD in an adult psychiatric population comprised of individuals with diverse and complex disorders, where symptoms of ADHD may be obscured by other symptoms? Our hypothesis was that neuromuscular dysregulation would significantly predict the presence of ADHD, but not affective disorders, anxiety disorders, or personality disorders.

## Methods

### Design

A naturalistic cross section study integrated in clinical practice “as usual.” Participants recruited from a general psychiatry outpatient clinic in a rural district of Norway were examined with regard to neuromuscular regulation difficulties.

### Inclusion Criteria

Patients remitted for diagnostic assessment

### Exclusion Criteria

Schizophrenia, other psychotic disorder, ongoing drug abuse, rheumatic-, orthopedic- or neurological disorders, and drugs that may affect motor function.

### Diagnostic Assessment

Diagnostic assessment was done by the clinician (physician or clinical psychologist), as part of their routine assessment, including medical history and following structured interviews:

*The Mini International Neuropsychiatric Interview Version 5.0.0 (MINI-plus)* (Palma-Álvarez et al., 2023; Sheehan et al., 1998) is a widely used, structured screening interview for multiple DSM-IV-TR psychiatric disorders “designed to meet the need for a short but accurate structured psychiatric interview for multicenter clinical trials and epidemiology studies” (Sheehan et al., 1998). The M.I.N.I. plus version has a module for Adult ADHD, which requires that respondents endorse at least 6/10 childhood symptoms (four inattention symptoms, three hyperactivity/impulsive symptoms, three behavior symptoms not included in the DSM-IV and ICD-10, and 9/14 adult symptoms (five inattention symptoms, three hyperactivity/impulsivity symptoms, three behavior symptoms not included in DSM-IV and ICD-10). If less than 6 childhood symptoms, or no symptoms prior to age 7 years, the instruction is to skip assessment of adult symptoms. The MINI-plus ADHD symptoms are not identical to either the ICD-10 or the DSM-IV ADHD symptoms. Excellent specificity, but less sensitivity and only moderate interrater reliability is reported for the MINI-plus ADHD module (Palma-Álvarez et al., 2023).

*The Iowa Personality Disorder Screen (IPDS)* (Langbehn et al., 1999) is a brief, sensitive screening interview for personality disorders.

Following questionnaires and interviews were applied as appropriate in the clinic:

*The Structured clinical interview for personality disorders (SCID-II)* (First, 1997) is designed to provide categorical assessment of different personality disorders.

*The Diagnostic Interview for assessment of ADHD in Adults 2.0 (DIVA-2.0)* (J. J. S. Kooij & Francken, 2009) is a semi-structured interview covering the nine inattention and nine hyperactivity/impulsivity ADHD symptoms in both childhood and adulthood, and also impairments in everyday life associated with the symptoms. The DIVA-2.0 is a reliable and valid tool for assessing and diagnosing adult ADHD (Ramos-Quiroga et al., 2019).

*The Adult ADHD Self-Report Scale (ASRS)* (Kessler et al., 2005) was developed in conjunction with revision of the WHO Composite International Diagnostic Interview. The ASRS includes nine inattention questions and nine hyperactivity/ impulsivity questions about DSM-IV Criterion A symptoms of ADHD, graded from 0 (*never*) to 4 (*very often*).

Additional questionnaires used for the present study were:

*The Autism Spectrum Quotient* (*AQ*) (Baron-Cohen et al., 2001) is a self-report questionnaire for autistic traits associated in adults with normal intelligence. It comprises 50 questions, assessing social skill, attention switching, and imagination. A score of 32 or more has been shown to indicate clinically significant levels of autistic traits (Wheelwright et al., 2010).

*The Meta Cognitions Questionnaire* (*MCQ*) (Cartwright-Hatton & Wells, 1997) measures anxiety-specific thinking like beliefs about worrying and intrusive thoughts, proneness to worry, and obsessional symptoms, and has demonstrated good psychometric properties.

### Diagnoses Included

#### Attention deficit hyperactivity disorder

Both the combined and predominantly inattentive type were included (1). ADHD was categorized as yes or no, regardless of subtype.

#### Affective Disorder

Included diagnoses were recurrent depression, depressive episode, dysthymia, and bipolar spectrum disorders. Affective disorder was categorized as yes or no, regardless of whether the patient had one or more comorbid affective disorder diagnoses.

#### Anxiety Disorder

Diagnoses included were panic disorder, agoraphobia, social phobia, generalized anxiety disorder, posttraumatic stress disorder, and obsessive-compulsive disorder. Anxiety disorder was categorized as yes or no, regardless of whether the patient had one or more comorbid anxiety diagnoses.

#### Personality Disorder

Personality disorder was categorized as yes or no, regardless of whether the patient had one or more comorbid personality disorder diagnoses.

### Assessment of Neuromuscular Regulation Problems

*The Motor Function Neurological Assessment* (*MFNU*) (Stray et al., 2006) was designed to detect clinically observed motor regulation problems in ADHD, in particular *neuromuscular inhibition problems* and *increased muscle tone*. The MFNU consists of 16 subtests with a qualitatively based scoring system. The scores 0, 1, or 2 are reflecting three scoring categories (0 = *Normal function*, 1 = *Moderate problems*, 2 = *Severe problems*), with a maximum summed problem score (MFNU-TS) = 32 (Stray et al., 2006). The internal consistency of the MFNU-scale (MFNU-TS) is very high (Cronbach’s alpha = .98) (Stray, 2009). Interrater agreement between physiotherapists who had received supervision on the use of the test, showed a high to very high rater reliability for each sub-test (Kappa ranging from .67 to 1.00). An Intraclass Correlation (ICC) of consistency calculated on the MFNU-TS for raters with limited experience in the use of MFNU, showed an average ICC of 0.99 (95% confidence interval [0.98, 1.00]), *p* < .001 (Stray, 2009). The use of the MFNU in clinic dates back to the 1990s. The test makes few demands on equipment, space, and time, and the tasks require minimal motor skills and concentration (Stray, 2009). In this study the MFNU was administrated and rated by a trained physiotherapist.

## Statistics

Pre-study power analysis indicated a required sample size of 10 and 60 participants in the two groups (no-ADHD and ADHD). Statistical analyses were performed using Statistical Package for Social Sciences (SPSS), version 29. Two-sided alpha levels of *p* < .05 were considered statistically significant. Sample characteristics were analyzed using student *t*-test, chi-square or Fisher’s exact test, and correlation analyses (Pearson *r* or Spearman rho), as appropriate. Binary logistic regression was performed to assess the impact of MFNU-TS on the diagnostic categories ADHD, affective disorder, anxiety disorder and personality disorder. Due to limited cases in each category, the analyses were performed with a model containing only one independent variable (MFNU sum score).

Sensitivity and specificity for ADHD diagnoses at different MFNU scores were explored using the Receiver Operation Characteristic curve and corresponding Area Under the Curve (AUC). AUC ranges in value from 0 (100% wrong) to 1 (100 correct). To be diagnostically meaningful, the lower 95% confidence interval value of the AUC must be >0.5 (Nahm, 2022). We used Swets (1988) recommendations for evaluating AUC values: <0.70: poor; 0.70 to 0.79: fair; 0.80 to 0.89: good; and 0.90 to 1.00: excellent. Youden’s index (sensitivity + specificity ÷ 1) (Youden, 1950) was calculated as a proposed measure of optimal cut-off value for ADHD diagnosis. Participants with significant autistic traits (AQ sum score ≥ 32) were excluded from analyses regarding MFNU, to reduce the confounding effect of autistic traits.

## Ethics

Ethics approval and consent to participate in the study was performed in accordance with the Declaration of Helsinki, and approved by the Regional Ethics Committee of Southern Norway and the Data protection controller of Sørlandet Hospital (2014/1231). Written informed consent to participate was obtained from each participant. Ethics approval and consent to participate in the study were performed in accordance with the Declaration of Helsinki.

## Results

### Participants

**Figure 1.**
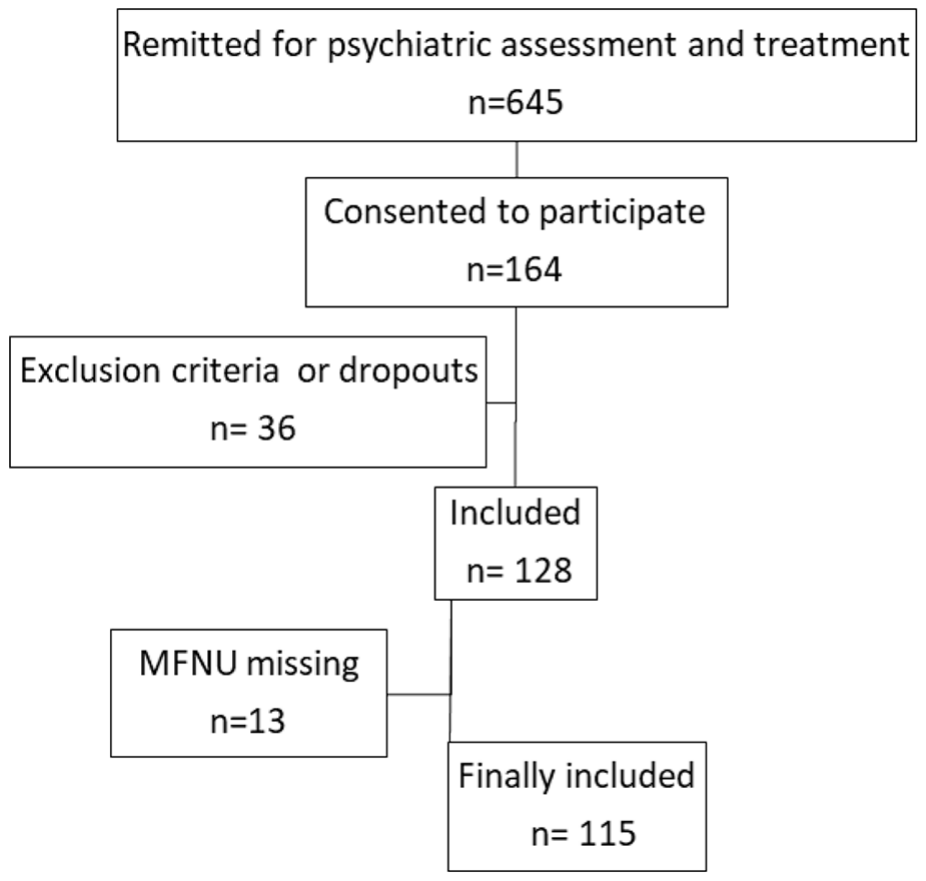
Flowchart inclusion of participants.

Figure 1 shows the flow chart of the study.

Among the included 115 participants (age 18–66, all Caucasian), 91 fulfilled the MINI-plus and/or DIVA-2.0 criteria for ADHD (the “ADHD-group”). Nine of these participants had a prior ADHD diagnosis. The mean number of DIVA-2.0 adult symptoms was 13.7; mean inattention and hyperactivity symptom sub-scores were 7.9 and 6.0, respectively. In the ADHD-group, 63.7% of the participants had comorbid symptoms within the other diagnostic groups, 26.4% within two or more additional diagnostic groups. The ADHD-group included 60 participants with ADHD combined subtype and 31 participants with ADHD predominantly inattentive subtype, these did not differ significantly regarding age, gender, and comorbidities, neither on mean MFNU-TS. The remaining 24 patients were included in the “NoADHD-group.” Within the NoADHD-group, 16 patients did reported disabling ADHD symptoms, but under cut-off for ADHD diagnosis mostly due to less than six childhood symptoms on the MINI. These were assessed by the clinicians as not having ADHD (in this study referred to as having subthreshold ADHD-symptoms). Six participants (two in the ADHD-group and four in the NoADHD-group) had an AQ sum score equal to or above 32, indicating significant autistic traits. None of these was diagnosed with autism spectrum disorder.

Clinical characteristics for the ADHD-group and the NoADHD-group are reported in Table 1.

**Table 1.** Characteristics of the Sample.

	ADHD-group (*n* = 91)	NoADHD-group (*n* = 24)	ADHD-group vs. NoADHD-group
Variables	*N* (%)		χ^2^; *p*
Female	54 (59.3)	18 (75.0)	2.0; .158
Affective disorders^a^	27 (29.7)	12 (50.0)	3.5; .061
Anxiety disorders^b^	33 (36.3)	12 (50.0)	1.5; .22
Personality disorders^c^	15 (16.5)	4 (16.7)	0.0; 1.0
	Mean (*SD*)		*t; p; d*
Age	33.0 (9.9)	32.0 (11.6)	0.4; .674
ASRS^d^	45.3 (10.6)	28.9 (8.4)	5.6; <.001
AQ^e^	20.6 (6.1)	19.9 (9.1)	0.3; .739
MCQ^f^	62.4 (13.8)	61.9 (14.6)	0.2; .875
MFNU-TS^g,h^	22.8 (2.3)	19.1 (6.7)	2.4; .025; 0.79
MFNU-TS-ex^g,h,i^	22.8 (2.3)	12.2 (7.1)	5.7; <.001; 2.40

*Note.* χ^2^ = Chi square or Fisher exact test, as appropriate; *t* = Student *t*-test; *d* = Cohen’s *d* effect size (small: 0.2, medium: 0.5, large: 0.8).

a Depression (*n* = 29, 22.7 %), Dysthymia (*n* = 13, 10.2 %), Bipolar spectrum (*n* = 10, 7.8 %, including one bipolar 1).

b Agoraphobia and panic disorder (*n* = 15, 11.7 %), Social Phobia (*n* = 17, 13.3 %), generalized anxiety disorder (*n* = 19, 14.8 %), posttraumatic stress disorder (*n* = 19, 14.8 %), obsessive-compulsive disorder (*n* = 6, 4.7 %).

c Borderline (*n* = 6, 4.7 %), Cluster C (*n* = 5, 3.9 %), mixed/unspecified (*n* = 11, 8.6 %).

d The Adult ADHD Self-Report Scale. Missing: 10 (11 %) in the ADHD-group, 9 (37.5 %) in the NoADHD group. Mean ASRS did not differ between those with and without subthreshold ADHD symptoms in the NoADHD-group.

e The Autism Spectrum Quotient. Missing: 14 (15.4 %) in the ADHD-group, 2 (8.3 %) in the NoADHD-group.

f The Meta Cognitions Questionnaire. Missing: 11 (12.1 %) in the ADHD-group, 4 (16.7 %) in the NoADHD-group.

g The Motor Function Neurological Assessment Total Score.

h Excluding six participants with significant autistic traits.

i Excluding from the No-ADHD group those with subthreshold ADHD symptoms.

## Delayed Diagnosis and Long-Term Outcome

Mean number of years from first contact with mental health service to ADHD diagnosis and treatment was 7.3 years, and was significantly correlated with “not being working or studying” (rho = .313, *p* = .004), and number of comorbid psychiatric diagnoses within the included diagnostic categories (rho = .375, *p* < .001), but not with age (rho = 149, *p* = .170) or gender (rho = .009, *p* = .934).

## MFNU Results

There were significant differences between the ADHD-group and the NoADHD-group regarding neuromuscular regulation problems (mean MFNU-TS [standard deviation] 22.8 [4.2] vs. 19.8 [6.4], *t* = 2.2; *p* = .036). When excluding from the NoADHD-group those 16 patients with disabling ADHD symptoms under cut-off for ADHD diagnosis, the mean MFNU-TS in the NoADHD-group declined (15.5 [8.6]). The mean MFNU-TS for those 16 patients with subthreshold ADHD symptoms was 22.0 (13–28). When excluding from the sample the six participants with significant autistic traits, the differences between the groups were more significant (Table 1).

In the sample as a whole, MFNU-TS was significantly correlated with the self-reported levels of ADHD symptoms, that is the ASRS total score (*r* = .284, *p* = .009), and the ASRS hyperactivity/impulsivity sub score (*r* = .303, *p* = .003), but not with the ASRS inattention sub score (*r* = .165, *p* = .063). The MFNU-TS was also significantly correlated with self-reported levels of autistic traits (the AQ sum score, rho = .266, *p* = .008), but not with self-reported level of anxiety-specific thinking (MCQ sum score, rho = .077, *p* = .445) age (*r* = .063, *p* = .500) or gender (rho = .053, *p* = .576).

## Effect of MFNU Total Score on Diagnostic Category

The results of the binary logistic regression analyses showed that MFNU-TS was a significant predictor of ADHD diagnosis only (see Table 2). Analyzing the ADHD subtypes separately had no effect on the result.

**Table 2.** Effect of MFNUs on Diagnostic Category, Binary Logistic Regression.

Dependent variable	*n*	Wald (*df* 1)	Odds ratio [95% confidence interval]	*p*	Nagelknerke *R*
Diagnosed ADHD^a^	109	8.0	1.15 [1.04, 1.27]	.005	.125
Diagnosed ADHD^a,b^	93	12.1	1.40 [1.16, 1.68]	<.001	.482
Affective disorder^a^	109	0.6	1.04 [0.95, 1.13]	.430	.008
Anxiety disorder^a^	109	0.4	0.97 [0.90, 1.05]	.509	.005
Personality disorder^a^	109	2.3	1.10 [0.97, 1.25]	.131	.041

a Excluding six participant with significant autistic traits.

b Excluding those with subthreshold ADHD symptoms from the No-ADHD-group.

## Diagnostic Accuracy

The ROC curve showed good to excellent accuracy for MFNU-TS in detecting ADHD, when excluding from the sample those with AQ score ≥ 32 and subthreshold ADHD symptoms (AUC = 0.90, *p* = .001), but rather poor diagnostic accuracy (though statistically significant) when including those with subthreshold ADHD symptoms (AUC = 0.66, *p* = .030).

Sensitivity and specificity, and likelihood ratios for ADHD diagnosis at different MFNU-TS are shown in Table 3. A MFNU-TS cut-off score of 13.5 yielded a near 98% sensitivity for ADHD diagnosis, both when including and excluding those with subthreshold ADHD symptoms. Including those with subthreshold ADHD symptoms yielded a rather low specificity at this cut-off score, and no clear cut Youden Index was revealed. When excluding those with subthreshold ADHD symptoms from the NoADHD-group, the specificity doubled, yielding the best Youden Index at MFNU-TS of 13.5.

**Table 3.** Group-Level Statistics for the MFNU-TS, ADHD Versus no ADHD.

MFNU-TS ≥	ADHD-group vs. NoADHD-group (*n* = 109)^a^			ADHD-group vs. NoADHD-group^b^ (*n* = 93)		
	*Se*	*Sp*	Y.I.	*Se*	*Sp*	Y.I.
7.0	1.00	0.10	0.10	1.00	0.33	0.33
10.0	0.99	0.15	0.14	0.99	0.50	0.49
13.5	0.98	0.25	0.23	0.98	0.77	0.74
17.5	0.93	0.30	0.23	0.93	0.67	0.60
19.5	0.78	0.40	0.18	0.78	0.83	0.61
22.5	0.54	0.65	0.19	0.54	1.00	0.54
26.5	0.20	0.95	0.15			
28.5	0.10	1.00	0.10			

*Note*. Se = sensitivity; Sp = specificity; Y.I = Youden index (sensitivity + specificity − 1).

a Excluding six participant with significant autistic traits.

b Excluding those with subthreshold ADHD symptoms from the No-ADHD-group.

## Discussion

This is, to the best of our knowledge, the first study to explore the utility of assessment of neuromuscular dysregulation in psychiatric differential diagnosis. As hypothesized, neuromuscular dysregulation predicted ADHD diagnosis, but not affective diagnosis, anxiety diagnosis, or personality disorder diagnosis. The results indicates that neuromuscular dysregulation may be a clinical sign of ADHD. This may in turn facilitate earlier diagnosis and treatment of ADHD in patients with a complex array of symptoms, thereby mitigating possible adverse consequences of misdiagnosis and delayed diagnosis (Libutzki et al., 2020). In line with previous studies, we found that a delayed ADHD diagnosis and treatment, was associated with negative long-term outcome (Libutzki et al., 2020; Shaw et al., 2012), in forms of increased comorbidity and not working or studying.

### Diagnostic accuracy

In our study, a MFNU-TS equal to or higher than 13.5 accurately identified participants with ADHD diagnosis. The specificity for ADHD diagnosis at MFNU-TS below 13.5 was low when including those with subthreshold ADHD symptoms but tripled when excluding these patients. This cut-off score also yielded the maximum Youden index. The cut-off level of MFNU-TS with the best balance between sensitivity and specificity for ADHD diagnosis, as compared to other psychiatric diagnoses, is clinically important. Where to set the cut-off, depends on what you consider as most important—not to overlook those with possible ADHD, or avoid assessing for ADHD persons with a low probability for the disorder. Given the poor long-term outcome for ADHD when left untreated, we argue that a high sensitivity is more important than a high specificity regarding ADHD diagnosis. Given that ADHD is a dimensional disorder with a significant hereditary component (Faraone et al., 2021; Katzman et al., 2017), subsyndromal ADHD or familial traits might influence neuromuscular dysregulation in participants without a formal ADHD diagnosis. Notably, 16 patients from the NoADHD-group reported substantial ADHD-related symptoms, albeit below the diagnostic threshold for ADHD. The mean MFNU-TS for these 16 patients aligned closely with the MFNU-TS results from the ADHD-group and was markedly elevated compared to the rest of the NoADHD-group, despite similar mean ASRS score as the rest of the NoADHD-group. Taken together, this suggests the possibility that some of these individuals might have had undiagnosed comorbid ADHD, underscoring the necessity for a thorough ADHD assessment also in cases with subthreshold symptoms of ADHD. According to our results, a MFNU-TS of 13 - 14 seems to be a significant indicator of the need for a more thorough ADHD-assessment.

### Autistic traits

In our study, we found a positive correlation between neuromuscular dysregulation and self-reported symptoms of ADHD (ASRS) as well as autistic traits (AQ score). The correlation with autistic traits is not surprising given the frequent comorbidity and overlapping symptoms between autism spectrum disorders (ASD) and ADHD, including motor problems (Ming et al., 2007). Frequently reported motor problems in high functioning ASD are deficiencies in motor learning, impaired movement performance, inability to execute a sequence of actions, motor skill problems related to different visual sensitivity to the movement and postural responsivity to the optic flow (Faridi & Khosrowabadi, 2017). The motor problems of ASD are probably functionally different from what we have found in ADHD. It is highly likely, however, that the ASD motor problems may interfere with performance on the MFNU.

### Anxiety disorders

We found no significant correlation between MFNU-TS and self-reported anxiety-specific cognition (MCQ score). Restlessness and concentration problems are common in both anxiety disorders and ADHD, whereas preoccupation with worrying thoughts is a core symptom in anxiety disorders only. The absence of correlation between MCQ score and MFNU-TS suggests that this kind of neuromuscular dysregulation is not involved in anxiety disorders, even though other motor issues like balance and coordination problems related to cerebellar anomalies have been reported in chronic anxiety states (Baldacara et al., 2008). Such cerebellar deviations in anxiety disorders may be attributable to comorbid conditions given the high rates of anxiety disorders in individuals with ADHD (Sobanski, 2006), or to diagnostic inaccuracies, in that prolonged bodily manifestations of anxiety can be easily confused with ADHD-associated restlessness (J. J. Kooij et al., 2012).

### Relation to cognitive deficits

It has been argued that motor regulation problems in ADHD are mediated by attention or arousal deficits, and that the amelioration of motor problems following stimulant treatment is proportional to the amelioration of attention or arousal difficulties (Brossard-Racine et al., 2011; Egeland et al., 2012). Although inattention and hypoarousal may indeed affect motor performance, we find it implausible that neurocognitive challenges could exert a significant influence on resting muscle tone or automated flexor-extensor interactions (Stray et al., 2015).

In our study, the MFNU-TS correlated more strongly with hyperactivity/impulsivity symptoms than with attention deficit symptoms, contradicting that motor problems in ADHD are secondary to cognitive problems. Similar conclusions may be drawn from neuroimaging studies. Dysfunction of basal ganglia, which are responsible primarily for motor control, is a consistent finding in both childhood and adulthood ADHD (Kasparek et al., 2015; Schneider et al., 2006).

Moreover, the dopamine agonist MPH has been shown to attenuate both dysfunctions in fronto-striato-cerebellar networks (Noreika et al., 2013), and neuromuscular dysregulation in ADHD (Stray, Stray, Iversen, Ruud, & Ellertsen, 2009). Taken together, this suggests that neuromuscular dysregulation in ADHD is more likely associated with dopamine dysfunction, than with cognitive impairments.

### Clinical usefullness

Regardless of our finding of neuromuscular dysfunction as a predictor of ADHD when comparing common psychiatric conditions and comorbidities, such dysfunctions need not be specific to the ADHD-diagnosis. Neuromuscular dysregulation may also manifest in other neuropsychiatric disorders involving dopamine dysregulation (Speranza et al., 2021). This notion is supported by the correlation between MFNU-TS and self-reported autistic traits in our study. However, despite frequently co-occurring neural traits, neuroimaging studies of ASD, DCD, and/or ADHD suggests that each disorder has more unique than shared neural characteristics (Kangarani-Farahani et al., 2022). Thus, motor problems may manifest somewhat differently in these disorders, in ADHD as neuromuscular regulation and inhibition problems, in DCD as poor motor skills and coordination problems, and in ASD as more complex motor performance problems.

Future research with a more balanced sample of various neuropsychiatric and neurodevelopmental disorders seems essential to explore if neuromuscular dysregulation as could be considered as a unique predictor of ADHD.

Besides its possible diagnostic value, neuromuscular assessment may have implication for treatment planning. A high MFNU-TS could indicate a positive response to methylphenidate for core ADHD symptoms (Stray et al., 2010). Additionally, identifying neuromuscular regulation issues could help patients with ADHD in the management of muscular stiffness, aches, and fatigue commonly associated with the disorder (Ghanizadeh, 2010; Mundal et al., 2023; Stray et al., 2013).

### Study Limitations

A relatively small sample size, a lower-than-desired participation rate, and a small number of participants without an ADHD diagnosis, are serious limitations to the generalizability of our findings. Low participation is a general problem in psychiatric research, though (Bixo et al., 2021).

The predominance of ADHD in our sample may reflect that persons with ADHD symptoms were particularly interested in the study, as ADHD was described as the major topic in the written information about the study. Furthermore, ADHD is notably prevalent in Northern Europe, particularly in the catchment area of the present study (Deberdt et al., 2015; Suren et al., 2013). Still, a significant portion of our sample had diagnoses other than ADHD, which were not linked with MFNU scores.

The methodology we adopted, a “naturalistic cross-sectional study integrated into routine clinical practice,” has inherent shortcomings. Specifically, smaller clinical groups are likely underrepresented in limited samples. Since assessment for comorbid DCD is not a standard procedure in adult psychiatry, none of the participants underwent such evaluation, potentially influencing the MFNU score. DCD would particularly be expected to affect the scores of the MFNU-subtests involving motor coordination and balance (e.g., the dynamic balance and overflow tests). However, most of the subtests of MFNU were designed not to be demanding on motor skills, and mainly involve tasks that are not influenced by training or age (Stray et al., 2006).

The larger female representation in our study aligns with other research from adult outpatient settings (Nylander et al., 2009) and may be due to heightened internalizing symptoms, leading to a more pronounced perceived need (Hinshaw et al., 2022). That said, the MFNU-TS scores showed no significant gender differences in our study.

To our knowledge, the MFNU is the only existing clinical tool for testing the specific neuromuscular regulation problems observed in ADHD. The test was not primarily developed for diagnostic or research purposes, and the use of subjective judgment in scoring of the MFNU might be seen as a shortcoming. Nonetheless, high internal consistency and construct validity of the MFNU-test items treated as a quantitative scale together with a high inter-rater reliability suggests that the instrument is statistically robust and useful as a research tool (Stray, 2009).

## Conclusion and Implications for Practice

Our findings suggest that assessment of neuromuscular dysregulation may contribute to identifying ADHD in psychiatric patients with a complex symptom picture overlapping with other psychiatric diagnoses. The identification of neuromuscular dysregulation problems could serve as a potentially valuable clinical sign of ADHD, supplementing the recognized behavioral and developmental indicators of the condition. Nonetheless, it is premature to assume that deficits in neuromuscular regulation are unique to ADHD. More well designed research with larger sample sizes and inclusion of a wider range of psychiatric and neurodevelopmental disorders are needed to reach a conclusion on this issue.

Our conclusions are based on the Motor Function Neurologic Assessment (MFNU) as an indicator of neuromuscular regulation problem. The test was not designed as a diagnostic instrument, but rather to systematically identify the neuromuscular problems observed in everyday life circumstances among individuals with ADHD (Stray et al., 2006). Nevertheless, the test has been used both as a clinical and as a research tool (Stray et al., 2015). In future research, tests that are more precisely tailored and operationalized for diagnostic and international research purposes would be beneficial for the further exploration of neuromuscular regulation issues and their association with ADHD.
